# *In Vivo* Two-Photon Imaging of Dendritic Spines in Marmoset Neocortex^1^,^2^,^3^

**DOI:** 10.1523/ENEURO.0019-15.2015

**Published:** 2015-09-17

**Authors:** Osamu Sadakane, Akiya Watakabe, Masanari Ohtsuka, Masafumi Takaji, Tetsuya Sasaki, Masatoshi Kasai, Tadashi Isa, Go Kato, Junichi Nabekura, Hiroaki Mizukami, Keiya Ozawa, Hiroshi Kawasaki, Tetsuo Yamamori

**Affiliations:** 1 Laboratory for Molecular Analysis of Higher Brain Function, Brain Science Institute, RIKEN, Wako, Saitama 351-0198, Japan; 2 Division of Brain Biology, National Institute for Basic Biology, Aichi 444-8585, Japan; 3 Department of Ultrastructural Research, National Center of Neurology and Psychiatry, National Institute of Neuroscience, Tokyo 187-8502, Japan; 4 Division of Behavioral Development, National Institute for Physiological Science, Aichi 444-8585, Japan; 5 Division of Homeostatic Development, National Institute for Physiological Science, Aichi 444-8585, Japan; 6 Division of Genetic Therapeutics, Center for Molecular Medicine, Jichi Medical University, Tochigi 329-0498, Japan; 7 Department of Biophysical Genetics, Graduate School of Medical Sciences, Kanazawa University, Ishikawa 920-8640, Japan

**Keywords:** dendrite, marmoset, neocortex, primate, spine

## Abstract

Two-photon microscopy in combination with a technique involving the artificial expression of fluorescent protein has enabled the direct observation of dendritic spines in living brains. However, the application of this method to primate brains has been hindered by the lack of appropriate labeling techniques for visualizing dendritic spines. Here, we developed an adeno-associated virus vector-based fluorescent protein expression system for visualizing dendritic spines *in vivo* in the marmoset neocortex. For the clear visualization of each spine, the expression of reporter fluorescent protein should be both sparse and strong. To fulfill these requirements, we amplified fluorescent signals using the tetracycline transactivator (tTA)–tetracycline-responsive element system and by titrating down the amount of Thy1S promoter-driven tTA for sparse expression. By this method, we were able to visualize dendritic spines in the marmoset cortex by two-photon microscopy *in vivo* and analyze the turnover of spines in the prefrontal cortex. Our results demonstrated that short spines in the marmoset cortex tend to change more frequently than long spines. The comparison of *in vivo* samples with fixed samples showed that we did not detect all existing spines by our method. Although we found glial cell proliferation, the damage of tissues caused by window construction was relatively small, judging from the comparison of spine length between samples with or without window construction. Our new labeling technique for two-photon imaging to visualize *in vivo* dendritic spines of the marmoset neocortex can be applicable to examining circuit reorganization and synaptic plasticity in primates.

## Significance Statement

Investigation of nonhuman primate brains is important for the understanding of the human brain. However, because of technical difficulties, several important methods that have been used in rodent studies are not available for primate studies. Two-photon imaging of dendritic spines has been used in rodent studies, which clarified the basis of neural circuit plasticity, but there has been no report of the application of this imaging method to primate brains. Therefore, in this study, we developed an adeno-associated virus vector-based fluorescent protein expression system for use in the studies of the marmoset neocortex. Our approach enabled the sparse yet strong expression of fluorescent protein in neurons. This labeling technique will be applicable to research into the circuit reorganization of primate brains.

## Introduction

Direct observation of fine neuronal morphologies such as dendritic spines in living brains has been made possible with techniques involving the expression of fluorescent protein in neurons of living animals in combination with two-photon microscopy. Many researchers prefer to use transgenic mouse lines such as Thy1-GFP and Thy1-YFP mice for their imaging studies of dendritic spines, because of the stable and strong expression of fluorescent protein in a subpopulation of neurons in these mouse lines (Feng et al., 2000; Grutzendler et al., 2002; Trachtenberg et al., 2002; Holtmaat et al., 2005; Zuo et al., 2005; Kim and Nabekura, 2011; Fu et al., 2012). The advancement of two-photon microscopy was also a key factor for the application of spine-imaging techniques to living animals (Denk et al., 1990; Denk and Svoboda, 1997). The long-wavelength light used in two-photon microscopy penetrates a specimen with less scattering than the short-wavelength light used in other conventional microscopy techniques such as confocal microscopy; thus, it is possible to observe signals from deep regions in a certain thick tissue.

Dendritic spine imaging by two-photon microscopy has been almost exclusively performed on rodent brains, and there have been only a few studies in which the brains of larger species such as primates and carnivores have been examined. One major reason for the lack of studies of such animals with larger brains is that there has been no standard method for *in vivo* imaging in these species. Transgenic monkey studies have been generated (Sasaki et al., 2009; Niu et al., 2010), but no primate model that strongly expresses fluorescent protein for observing signals *in vivo* has been established. Spine imaging of the primary visual cortex of ferrets using an expression system with a virus vector was reported (Yu et al., 2011). In the case of primates, one group studied the neuronal morphology in the primary visual cortex of macaque monkeys by *in vivo* imaging using virus expression systems (Stettler et al., 2006; Yamahachi et al., 2009), but their observation and analysis focused not on dendritic spines but on axonal structures. Using virus vector-based methods for the labeling of neurons to study their morphology is difficult because the high density of labeled neurons around the injection site also makes the background signal intensity high, thus making such methods unsuitable for the observation of the morphology of fine structures such as dendritic spines. Although dendritic spines in the primate cortex have been extensively analyzed in fixed samples by dye injection methods (Elston et al., 1999; Oga et al., 2013), there has been no report in which dendritic spines were imaged and analyzed *in vivo* in primate brains.

In this report, we present a method of *in vivo* imaging of dendritic spines in the marmoset neocortex. The marmoset was chosen as a model animal in our study because the flat surface of a marmoset brain is advantageous for studying the entire cortical region. We mainly had to overcome two technical problems in the *in vivo* visualization of dendritic spines in the marmoset neocortex. We needed a stronger expression of fluorescent protein because the marmoset brain is more opaque than the brains of smaller animals such as mice. In addition, we needed neurons to be sparsely labeled, because the dense expression obtained by the virus vector method usually causes a high background signal intensity. We, therefore, used an adeno-associated virus (AAV) expression system for the sparse and strong expression of the fluorescent protein in cortical neurons of marmosets, and by two-photon microscopy we were able to observe more clearly dendritic spines labeled by fluorescent proteins.

## Materials and Methods

### Animals

We used six marmosets (all males; body weight, 310–420 g; age, 13–22 months); five animals for *in vivo* imaging and one animal for dye injection. All the protocols used in this study were approved by the Institutional Animal Care and Use Committee of the National Institutes of Natural Sciences, Japan. The experiment was also conducted in accordance with the *Guide for the Care and Use of Laboratory Animals* of the U.S. National Institutes of Health.

### Plasmid construction and AAV preparation

**Figure 1.**
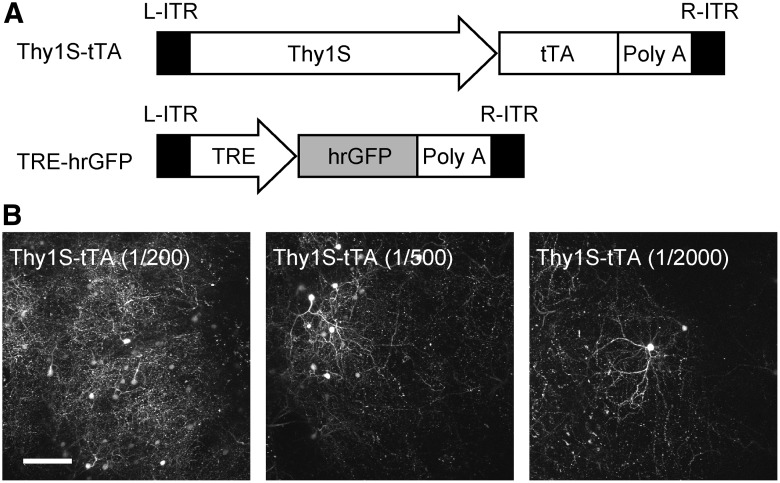
Thy1S promoter drives sparse expression of hrGFP in marmoset cortex. ***A***, Schematic illustration of virus constructs. ***B***, The expression of hrGFP at different concentrations of virus injection was imaged in fixed brain samples using two-photon microscopy. Left, Low concentration; middle, medium concentration; right, high concentration. Note the difference in labeled cell density at different concentrations. Maximum intensity projections of 71, 71, and 51 slices, respectively, for left, middle, and right panels at intervals of 5 µm. Scale bar, 100 µm.

The constructs used in this study are schematically shown in Figure 1*A*. The Thy1S promoter was cloned from pThy1S-GFP for the sparse labeling of cortical neurons as previously reported (Ako et al., 2011). Owing to capacity limitations of the AAV vector, we truncated ∼1.3 kb of the 5' region of the Thy1S promoter, which is reported to be nonessential for the activity of the promoter (Vidal et al., 1990; Caroni, 1997). The plasmid AAV (pAAV):Thy1S-tetracyline transactivator (tTA) was constructed by subcloning the DNA fragments containing the truncated Thy1S promoter and tTA in pAAV-MCS (Agilent Technologies). The pAAV:tetracycline-responsive element (TRE)-humanized renilla GFP (hrGFP)-humanized renilla GFP (hrGFP) was constructed by replacing the TurboRed fluorescent protein (tRFP) sequence of AAV:TRE-tRFP (Watakabe et al., 2014) with hrGFP.

 The AAV vectors used in this study have capsids of serotype 1. They were produced in HEK 293 cells using a helper-virus-free system and were purified twice by CsCl_2_ density gradient centrifugation and titrated by quantitative PCR, as described previously (Konishi et al., 2008). The final preparations obtained were dialyzed against PBS and diluted as described in the Results section. To prevent adhesion of the AAV vector to glass micropipettes, Pluronic-F68 (Sigma-Aldrich) was added to the vector stock at 0.001%.

### Virus injection

The marmosets were treated by intramuscular injections of ketamine (20 mg/kg; Daiichi Sankyo) and xylazine (1 mg/kg; Bayer Health Care). Under deep anesthesia induced and maintained by isoflurane (1-2%) inhalation (Abbott Laboratories), the head of the animal was fixed to a stereotaxic apparatus. Pulse rate, O_2_ saturation (SpO_2_), and rectal temperature were continuously monitored. A small hole was drilled in the skull using a dental drill. To inject the viruses into the cortex, the dura was punctured using the tip of a 27 ga needle, through which a glass pipette was slowly inserted to a depth of 500 µm from the cortical surface. Approximately 0.5 µl of a viral solution was injected at a rate of 0.1 µl/min. For the imaging of the prefrontal cortex (PFC), our injections were targeted anteroposterior +18.5 mm, 2 mm to the right from the midline. Following the viral injection, the hole was filled with Spongel, an absorbable gelatin sponge (Astellas Pharma Inc.), and the scalp was sutured. Then the animal was returned to a cage and remained there until the imaging sessions started. To prevent infection, ampicillin (40 mg/kg; Meiji Seika Pharma) was administered intramuscularly. Carprofen (5 mg/kg; Pfizer) was administered intramuscularly as an analgesic and an anti-inflammatory agent. Ampicillin and carprofen were administered immediately after surgery and 2 subsequent days. We waited until an adequate level of gene expression was obtained, which took at least 2 weeks.

### *In vivo* imaging

**Figure 2.**
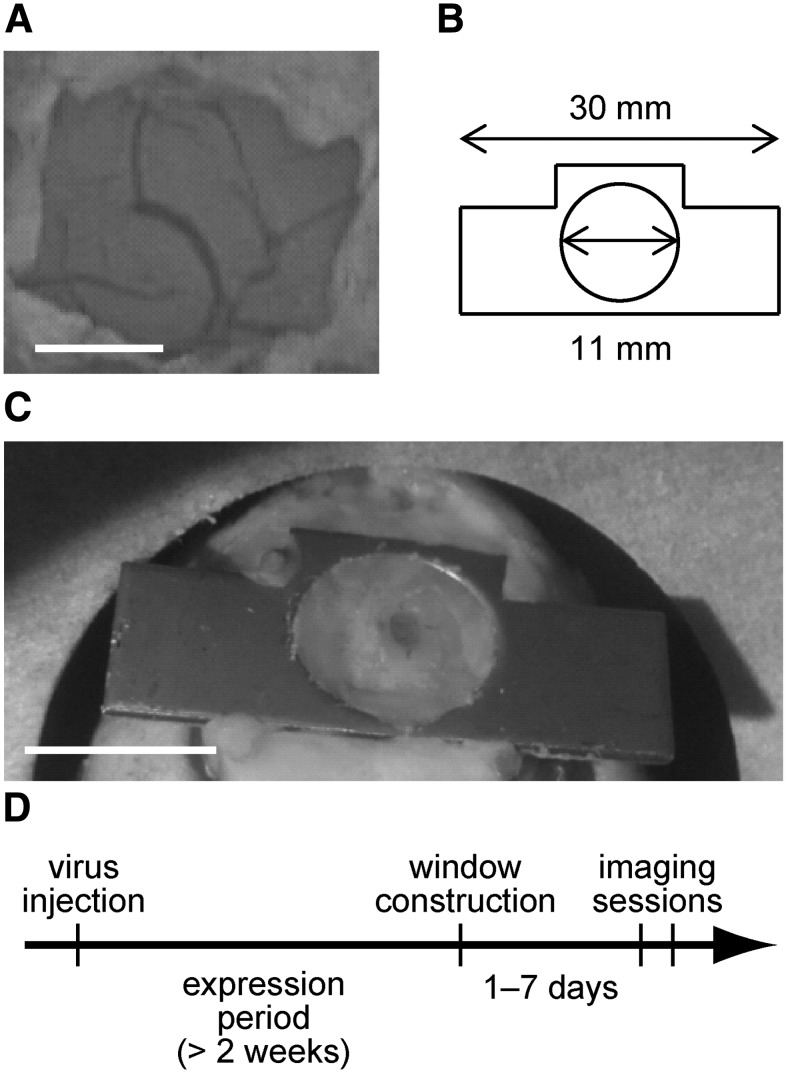
Construction of imaging window. ***A***, Craniotomy and durotomy over the target region around the somatosensory cortex. The exposed target region of the marmoset cortex is shown. Scale bar, 500 µm. ***B***, Illustration of the metal plate used in this study. **C**, Picture showing the metal plate for fixation, attached to the marmoset head. Scale bar, 10 mm. ***D***, Experimental schedule.

Before an imaging session, we constructed an imaging window on the head of the animal. The hole in the skull used for virus injection was expanded to a size of ∼2 × 3 mm^2^ using a dental drill, and part of the dura above the injection site was deflected and resected, yielding an incision ∼1 mm in diameter (Fig. 2*A*). A small coverglass ∼4 × 4 mm^2^ in size was fixed with dental cement on top of the skull, and the space between the coverglass and the cortex was filled with an agarose gel (1.5% in artificial CSF; type III-A; Sigma-Aldrich) to minimize vibration. A custom-made metal plate with a hole having an 11 mm inner diameter was glued to the skull (Figs. 2*B*,*C*). This plate was used to fix the head of the animal during the imaging sessions. The same antibiotic, analgesic, and anti-inflammatory agents as those used for the virus injections were administered immediately after window construction and on 2 subsequent days. We started the imaging sessions from 1 to 7 d after the imaging window construction (Fig. 2*D*).

*In vivo* two-photon imaging was performed using a FV1000MPE Multiphoton Laser Scanning Microscope (Olympus) and a water immersion objective lens [25×; numerical aperture (NA), 1.05; Olympus]. Two-photon excitation (920 nm) was provided by a mode-locked Ti:Sapphire laser (MaiTai Deep See, Spectra-Physics). Fluorescence was detected using a multialkali photomultiplier tube (PMT) without any filter in front of the PMT. The regions with fluorescently labeled dendrites were identified without digital zoom (field of view, 508 × 508 µm^2^). Then we used an 8× digital zoom to acquire images of magnified sites including dendritic spines at a resolution of 0.124 × 0.124 × 0.2 µm^3^ (field of view, 63 × 63 µm^2^). These sites were identified the following day, and the images obtained on 2 consecutive days were analyzed. Imaged sites were scattered around a 508 × 508 µm^2^ region and were presumed to contain dendrites originating from different neurons. During imaging sessions, the marmosets were anesthetized with isoflurane (1-2%). Pulse rate, SpO_2_, and rectal temperature were continuously monitored.

### Dye injection

A marmoset was sedated with ketamine hydrochloride (25 mg/kg, i.m.; Daiichi Sankyo) and were overdosed with sodium pentobarbital (75 mg/kg, i.p.; Dainippon Sumitomo Pharma). The animal was perfused intracardially with 0.1 m potassium PBS, pH 7.2, followed by 4% paraformaldehyde (Merck) in 0.1 m PB. A block of tissue was excised from the PFC. Coronal slices of 250 µm thickness were prepared from the block. Slices were incubated in DAPI (Sigma-Aldrich) solution to visualize the cell bodies. Pyramidal cells were individually injected with 10 mm Alexa Fluor 568 (Alexa Fluor 568 Hydrazide in 200 mm KCl, A-10441; Thermo Fisher Scientific) under visual guidance with a triple-band fluorescence filter (Semrock). The dye-injected neurons were imaged using a Leica SP-8 confocal laser scanning microscope and a water-immersion lens (63×; NA, 1.2; Leica) at a resolution of 0.045 × 0.045 × 0.336 µm^3^.

### Immunohistochemical analysis

Marmosets were sedated with ketamine hydrochloride (25 mg/kg, i.m.; Daiichi Sankyo) and overdosed with sodium pentobarbital (75 mg/kg, i.p.; Dainippon Sumitomo Pharma). The animals were perfused transcardially with 0.9% NaCl and then fixed with 4% paraformaldehyde in 0.1 m phosphate buffer, pH 7.4. Brain samples were cryoprotected with 30% sucrose/0.1 m phosphate buffer, pH 7.0, and sectioned at thicknesses of 40–50 µm using a cryostat. For immunofluorescence analysis, the sections were treated with 80% methanol/20% dimethyl sulfoxide solution (Dent’s solution) for >30 min, blocked with 10% fetal bovine serum, 2% bovine serum albumin, and 0.5% Triton X-100 in TBS, pH 7.4, and then incubated overnight with a primary antibody to hrGFP (1:4000; Vitality hrGFP rabbit polyclonal antibody; catalog #240141, Agilent Technologies) at 4°C. After incubation with a Cy2-conjugated secondary antibody (anti-rabbit Cy2; 1:1000; Jackson ImmunoResearch), the sections were counterstained with Hoechst 33342 (1:2000; Molecular Probes). For immunostaining with anti-GFAP and Iba1 antibodies, rabbit polyclonal antibodies from Abcam (catalog #AB7260) and Wako (catalog #019-19741) were used, respectively, as the primary antibodies, followed by staining with Cy3-conjugated anti-rabbit IgG (1:1000; Jackson ImmunoResearch).

### Image analysis

We used ImageJ (National Institutes of Health), Neurolucida (MBF Bioscience), and custom-made software written on MATLAB (R2009a; MathWorks) for our image analysis. Images were processed with a median filter (2.0 pixel radius) to reduce noise. Dendrites and spines were traced and marked manually in a three-dimensional space. The loss or gain rate of dendritic spines was calculated as the percentage of spines that appeared or disappeared on day 1, relative to the total number of spines on day 0. The length of spines was measured from the tip of the spine to the interface with the dendritic stalk (Ji et al., 2010).

### Statistical analysis

All statistical analyses were performed using R (R Development Core Team, 2012). We used the Wilcoxon rank sum test to compare between groups, and we corrected for multiple comparisons when required. Differences were considered to be significant at *p* < 0.05. Measurements are reported as the mean and SD. The values of statistical power were calculated using G*Power (http://www.gpower.hhu.de/en.html) and are presented in Table 1. Experimental animals were randomly assigned to *in vivo* or *ex vivo* conditions.

**Table 1:** Statistical table

	Data structure	Type of test	Power
a	Normality not assumed	Wilcoxon rank sum test	0.51
b	Normality not assumed	Wilcoxon rank sum test with Holm correction	1.00
c	Normality not assumed	Wilcoxon rank sum test with Holm correction	0.97
d	Normality not assumed	Wilcoxon rank sum test with Holm correction	1.00
e	Normality not assumed	Wilcoxon rank sum test with Holm correction	0.79
f	Normality not assumed	Wilcoxon rank sum test with Holm correction	0.51
g	Normality not assumed	Wilcoxon rank sum test with Holm correction	1.00

## Results

### Sparse and strong expression system for spine visualization

To observe the dendritic spines of neurons in the marmoset cortex *in vivo*, there are the following two requirements: strong and sparse expression of fluorescent protein. The scattering of light in living tissues prevents weak signals from being captured by the detector, particularly in the marmoset brain, which is more opaque than the mouse brain; thus, a strong expression is required. Moreover, to observe fine structures such as spines, the expression of fluorescent protein should be sufficiently strong to adequately label these fine structures. Sparse expression is also required to reduce the intensity of background signals. Even when the expression level of fluorescent protein in each neuron is sufficiently strong, a dense expression of fluorescent protein in neighboring neuronal structures such as dendrites and axons makes the intensity of background signals high, thus preventing the clear observation of dendritic spines.

Our strategy to achieve the requirements described above was to combine the tTA–TRE system and Thy1S promoter (Ako et al., 2011) using two virus vectors. We constructed two AAV vectors: one had the tTA component under the control of the Thy1S promoter (AAV:Thy1S-tTA) and the other had hrGFP under the control of TRE (AAV:TRE-hrGFP). Figure 1*A* shows a schematic drawing of our virus constructs. Ako et al. (2011) developed the Thy1S promoter to sparsely label the fine structures of neurons in the mouse neocortex. When expressed by electroporation in the mouse neocortex, the Thy1S promoter drives the gene expression only in a small number of pyramidal neurons in layers 2/3 and 5. In our expression system, the tTA–TRE system was driven only in Thy1S-positive cells. Hioki et al. (2009) showed that the tTA–TRE system is effective in amplifying transgene expression. The tTA–TRE system has two components, one is tTA and the other is TRE. When tTA binds to the TRE component, the transcription of hrGFP that is under the control of the TRE component is strongly activated.

We injected a mixture of these virus vectors into the marmoset neocortex and first examined the expression in fixed brain samples (Fig. 1*B*). We were interested in the density of hrGFP-positive neurons and the visibility of each dendrite. In our preliminary mouse experiment, we found that maintaining the concentration of AAV:TRE-hrGFP at 4.6 × 10^12^ vector genomes (vg)/ml and reducing the concentration of AAV:Thy1S-tTA to ∼1/500 of the TRE vector (8.8 × 10^9^ vg/ml) leads to the sparse but strong expression of the fluorescent protein (data not shown). On the basis of this finding, we tested various concentrations of AAV:Thy1S-tTA relative to that of AAV:TRE-hrGFP in marmosets. At an AAV:Thy1S-tTA to AAV:TRE-hrGFP concentration ratio of 1:200, the number of hrGFP-positive neurons was relatively high (Fig. 1*B*, left). At a concentration ratio of 1:2000, the number of hrGFP-positive neurons was too small (Fig. 1*B*, right). An AAV:Thy1S-tTA to AAV:TRE-hrGFP concentration ratio of 1:500 yielded the most desirable signals in terms of intensity and density (Fig. 1*B*, middle).

We emphasize the importance of amplifying the expression level of hrGFP using the tTA–TRE system. Simply reducing the titer of the virus leads to a low expression level, thus making it difficult to observe spines *in vivo*. The combination of titration and amplification was required for the clear visualization of dendritic spines in the marmoset neocortex.

### *In vivo* visualization of dendritic spines in neocortex

**Figure 3.**
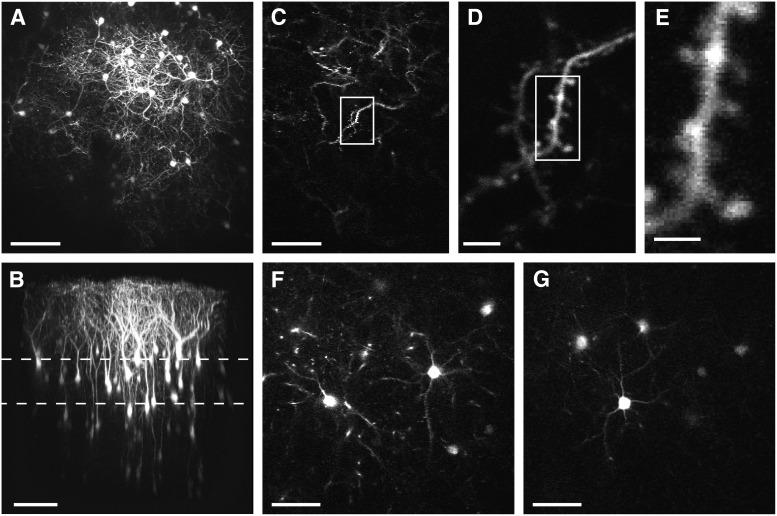
Dendritic spines imaged by *in vivo* two-photon microscopy. ***A***, Maximum intensity projection of the images acquired by *in vivo* two-photon imaging of marmoset cortex. *B*, Side view of three-dimensional reconstruction of the images of the same site shown in ***A***. The depths of the areas shown in ***F*** and ***G*** are indicated by dashed lines. ***C***, Image plane near pial surface. ***D***, Magnified image of the boxed area in ***C***. ***E***, Magnified image of boxed area in ***D*** showing dendritic spines. ***F***, Image plane at a depth of 220 µm showing soma and basal dendrites. ***G***, Image plane at a depth of 330 µm. Scale bars: ***A***, ***B***, 100 µm; ***C***, 50 μm; ***D***, 5 μm; ***E***, 2 μm; ***F***, ***G***, 50 µm.

To test the feasibility of our viral expression system *in vivo*, we injected the virus into the marmoset neocortex. After 2 weeks of an expression period, we acquired images from living animals under anesthesia induced by isoflurane. Figure 3 shows *in vivo* captured images. We were able to visualize each dendritic spine using our virus constructs (Fig. 3*C–E*). We observed the dendritic spines of apical dendrites located in layer 1. We were also able to observe the cell bodies located at a depth of ∼300 µm from the pia (Fig. 3*F*, at 220 µm; Fig. 3*G*, at 330 µm).

**Figure 4.**
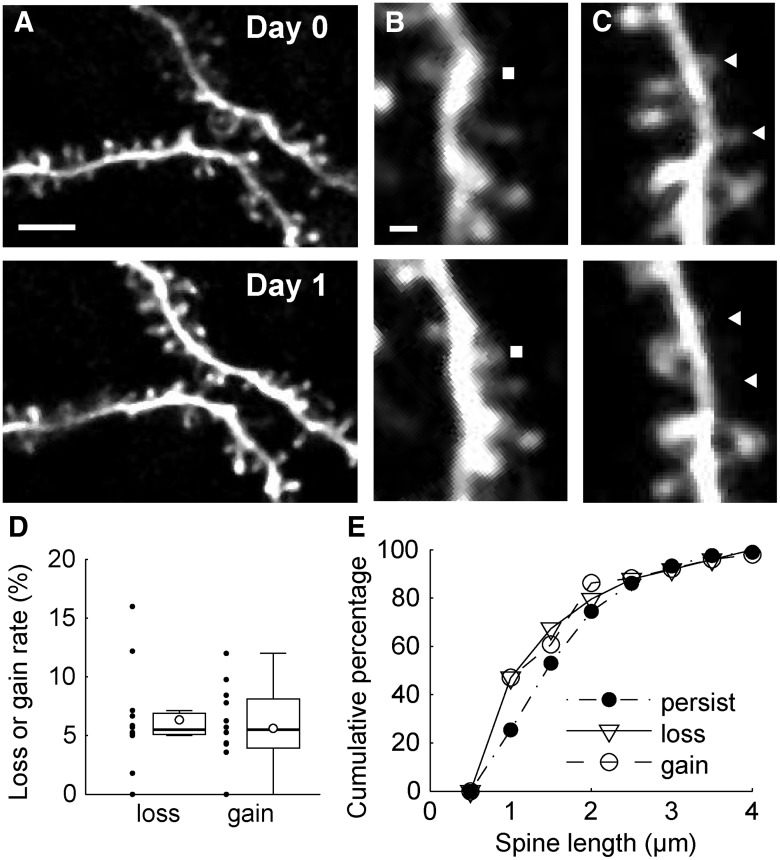
Time-lapse imaging of spines in prefrontal cortex. ***A***, The same dendritic regions in the prefrontal cortex were imaged at 24 h intervals. The top panel shows an image acquired on day 0 (7 d after craniotomy), and the bottom panel shows an image acquired on day 1. Scale bar, 5 µm. ***B***, The gained spines were identified by manual inspection of two images acquired at 24 h intervals. A filled rectangle indicates the position of an example of a gained spine. Scale bar, 1 µm. ***C***, The same as ***B*** for lost spines. Filled triangles indicate the positions of lost spines. ***D***, Box plots showing the spine turnover rate. The open circles in box plots indicate mean values. Black dots indicate values for each site. The whiskers extend to the largest and smallest values within 1.5 times the interquartile range. ***E***, Cumulative distributions of spine length in persisting, gained, and lost populations.

We then acquired images repeatedly from the same region over time. Figure 4 shows time-lapse images of the dorsolateral prefrontal cortex, presumably area 8B or 9. The images of the same region of the dendrites were taken over time at 24 h intervals. During these imaging sessions, the clarity of the imaging window was maintained. Because our samples contained a relatively small number of hrGFP-positive neurons per injected site, we were able to easily identify the same dendrite that we observed in the previous imaging session. The overall shapes of dendrites did not change over this imaging period (Fig. 4*A*, top and bottom). We marked each spine on the two images (day 0 and day 1) by comparing these images side by side, and identified the spines that were “gained” or “lost” during this time interval (Fig. 4*B*,*C*). In our experiments, we analyzed 779 spines (12 sites; 34 dendrites; 3 animals; total dendrite length, 2238 µm); of these spines, 51 were gained (mean across sites, 6.4%; SD, 4.2%) and 49 were lost (mean across sites, 5.6%; SD, 3.6%; Fig. 4*D*). The loss or gain rate at the 1 d interval observed in this study was similar to those in previous studies of layer 5 neurons of the somatosensory cortex of transgenic mice (∼12% in 3 d for both loss and gain; Kim and Nabekura, 2011) and layer 2/3 neurons of ferret V1 by the virus vector method (∼4% in 1 d for both loss and gain; Yu et al., 2011). We measured spine length by manual tracing using Neurolucida software, and examined the difference between the distribution of the spines that persisted and that of the spines that changed (gained and lost) over the period of imaging. We observed the tendency that the changed spines were shorter than those that persisted (Wilcoxon rank sum test, changed vs persisted, *p* = 0.0009^a^), which means that shorter spines tend to be gained or lost (Fig. 4*E*).

**Figure 5.**
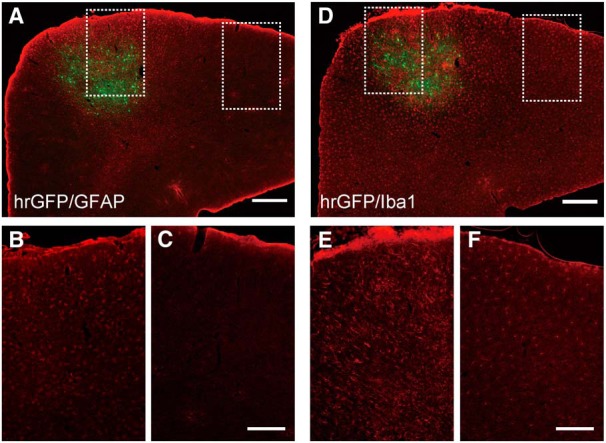
Activation of glial cells. ***A***, Confocal image of a sample immunohistochemically stained with anti-GFAP antibody. Scale bar, 500 µm. ***B***, Magnified image of left boxed area in ***A***, near the injection site. ***C***, Magnified image of the right boxed area in ***A***, distal from the injection site. Scale bar, 200 µm. ***D***, Confocal image of a sample immunohistochemically stained with anti-Iba1 antibody. Scale bar, 500 µm. ***E***, Magnified image of the left boxed area in ***C***, near the injection site. ***F***, Magnified image of the right boxed area in ***C***, distal from the injection site. Scale bar, 200 µm.

One of the concerns raised in the two-photon imaging of dendritic spines in mice studies was the activation of glial cells under invasive procedures. Because our methods in the marmoset neocortex include invasive procedures of virus injection and dura opening, we checked the activation of glial cells in our sample of the prefrontal cortex that was used for the *in vivo* imaging study. We observed the activation of both astrocytes (GFAP; Fig. 5*A–C*) and microglias (Iba1; Fig. 5*D–F*) around the injection site. This observation indicated that experimenters should carefully choose the experimental paradigm when applying the method presented in this article (see Discussion).

### Comparison between *in vivo* and fixed samples

**Figure 6.**
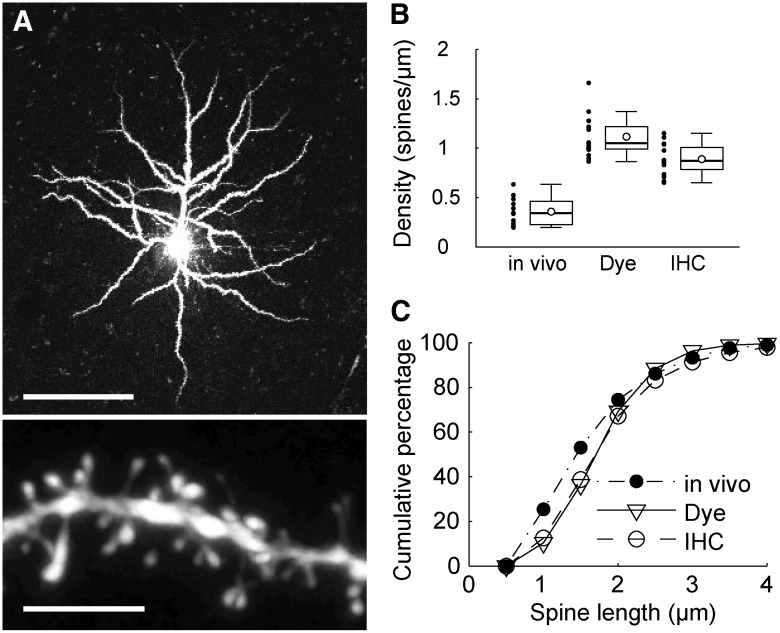
Comparison of dendritic spines between *in vivo* and *ex vivo* observations. ***A***, Confocal images of *ex vivo* dye-injected samples. Scale bars: top, 100 µm; bottom, 5 µm. ***B***, Box plots showing spine densities in *in vivo* and *ex vivo* populations. The open circles in box plots indicate mean values. Black dots indicate values for each site. The whiskers extend to the largest and smallest values within 1.5 times the interquartile range. ***C***, Spine densities of *in vivo*, dye-injected, and IHC samples. ***D***, Cumulative distribution of spine length under *in vivo* and *ex vivo* conditions.

To evaluate our *in vivo* two-photon microscope images of dendritic spines in the marmoset neocortex, we compared them with those of fixed samples. We wanted to determine whether we observed all existing spines or only a subpopulation of these spines. To clarify this point, we used the method that was used to identify the morphological differences of basal dendrites of pyramidal neurons in different cortical areas from various species, including marmosets and macaques (Elston et al., 1999; Oga et al., 2013). We injected the dye Alexa Fluor 568 into the neurons in the coronal sections of the marmoset prefrontal cortex, corresponding to the region we imaged under the *in vivo* condition, and observed the spines of apical dendrites of these dye-injected neurons under a confocal microscope (Fig. 6*A*; one animal; 15 sites; 22 dendrites; 1219 spines; total dendrite length, 1125 µm). Our injection labeled dendritic spines well in the distal region of apical dendrites. We quantified the density and shape of spines in our fixed samples by manual tracing using Neurolucida software, and we compared the results with our *in vivo* data. The density of spines under our *in vivo* condition (mean, 0.36 spines/µm; SD, 0.14) was significantly lower than that in dye-stained samples (mean, 1.12 spines/µm; SD, 0.21; Wilcoxon rank sum test with Holm correction, *p* = 0.00038^b^; Fig. 6*B*). The spines observed by *in vivo* two-photon imaging were shorter than those in dye-stained fixed tissues (Wilcoxon rank sum test with Holm correction, *p* = 7.6 × 10^−12c^; Fig. 6*C*). This comparison of results between *in vivo* two-photon imaging and dye staining of fixed samples indicated that the observation of dendritic spines by the *in vivo* imaging system still has certain limitations.

As for the causes of these limitations, there are two possibilities. The most likely possibility is that because of inefficient visualization, we underestimated the number of spines in *in vivo* imaging. The second possibility is that the damage caused by AAV injection decreased the number of spines. To determine which of these two possibilities is true, we immunohistochemically stained our samples used *in vivo* imaging with the antibody to hrGFP immunohistochemically stained [immunohistochemistory (IHC) samples], and then observed these fixed samples by confocal microscopy (two animals; 15 sites; 20 dendrites; 839 spines; total dendrite length, 951 µm). Although one may consider the comparison between the observation *in vivo* and the same dendritic segments identified in fixed condition, it was difficult for us, in a practical way, to identify the same dendritic segments in the more crowded fixed samples because immunohistochemical staining amplified the signals even in dendrites that express hrGFP weakly. Thus, here we compared the observation *in vivo* with the dendritic segments at the matching regions from the same samples. The spine density of IHC samples was much higher than that of samples in *in vivo* imaging (Wilcoxon rank sum test with Holm correction, *p* = 0.00038^d^; Fig. 6*B*), suggesting that we were not able to detect weaker signals under *in vivo* two-photon imaging. However, the spine density of IHC samples was lower than that of dye-stained fixed samples (Wilcoxon rank sum test with Holm correction, *p* = 0.0028^e^), which also suggests that there may have been some losses of spines owing to tissue damage, as mentioned above. However, the distribution of spine length of IHC samples largely overlapped with that of dye-stained fixed samples, but not with that of *in vivo* samples (Fig. 6*C*). This indicates that the window construction only mildly affected dendritic spines (Wilcoxon rank sum test with Holm correction; IHC vs dye, *p* = 0.64^f^; IHC vs *in vivo*, *p* = 2.2 × 10^−10g^).

## Discussion

In this study, we established a method of visualizing dendritic spines in the marmoset cortex by *in vivo* two-photon imaging using a virus expression system. To the best of our knowledge, this is the first demonstration of two-photon imaging of dendritic spines in the neocortex of a living primate.

### Merits of this method

By virus injection, we can control gene expression and monitor the morphology of transduced neurons without affecting other parts of the brain. Even though transgenic marmoset lines in which a specific neuronal population expresses fluorescent protein may be available for *in vivo* imaging in the near future, virus expression systems have their own merits. One advantage is the shorter time required for their preparation. Generating transgenic primate lines requires a much longer time. Another merit is region-specific manipulation. The use of the combination of transgenic animals and virus expression systems will be beneficial in future studies. For example, a specific cell type can be targeted by the transgenic insertion of an appropriate promoter, and the local expression of a gene may be induced by an appropriate virus expression system.

### Technical considerations

Our method presented in this article still has some limitations. First, there may have been tissue damage due to the procedures used in our study. The dura of the marmoset brain is more opaque than that of the mouse brain, so we dissected the dura to observe dendritic spines *in vivo* by two-photon imaging. This procedure may be more invasive than that used in mice. In mice, some researchers argued that even removing the skull could perturb the underlying brain tissues and make dendritic spines more unstable (Xu et al., 2007). Indeed, as shown in Figure 5, we observed the activation of glial cells around the injection site. Since a previous study in the primary visual cortex of the ferret (Yu et al., 2011) was conducted under conditions comparable to those in our study (using the Sindbis virus), and examined the activity-dependent morphological plasticity of dendritic spines by comparing active and inactive ocular dominance column, likewise our methods presented in this article may be applicable to studies that compare morphological plasticity in different conditions, such as the level of sensory input or the state of learning. Therefore, we believe that our method is an important technical improvement toward the understanding of the function of the primate neocortex. However, we have to further improve our methods so that the tissue will be less damaged. One possible improvement is to construct an imaging window that permits the longer imaging period, and allows waiting for time until the activity of glial cells subsides. Another improvement could be developing procedures to acquire images through the dura. As part of the process of developing the methods described in this article, we are now on the way to acquire images of axons through the intact dura of the marmoset cortex. In future studies, the improvement of the method for imaging dendritic spines through the dura will lead to less invasive imaging.

The second limitation is in detecting weak signals, as shown in Figure 6. Therefore, we need to amplify such signals or use a detector with high sensitivity.

### Importance of examining spines in brains of living primates

The majority of imaging studies of spines were performed in mice, because many molecular biological techniques are available for mice. Although experiments using mice are important, there are certain functions and structures that only primates have acquired during the course of their evolution. For example, the area specialization of the neocortex is far more evolved in primates than in rodents. In previous studies, the area-specific gene expression profiles in the primate neocortex were examined (Yamamori and Rockland, 2006; Yamamori, 2011; Bernard et al., 2012). Genes selectively expressed in association areas (Komatsu et al., 2005; Takaji et al., 2009; Sasaki et al., 2010) and the primary visual cortex (Takahata et al., 2006; Watakabe et al., 2009) were reported. Importantly, area-selective expressions of these genes are not observed in rodents (Yamamori, 2011). In relation to the gene expression patterns in different cortical areas, previous studies have shown that the density of spines significantly differs among different areas of the primate neocortex (Elston et al., 1999, 2005; Elston and Rockland, 2002). There is a smaller difference in the density of spines among different cortical areas in mice than in primates (Ballesteros-Yáñez et al., 2006). In addition, previous studies by *in vivo* two-photon imaging of spines in mice showed that there is no significant difference in morphological plasticity among different areas (Zuo et al., 2005). It is, therefore, of great interest to us to determine whether there is a difference in morphological plasticity among different areas in the primate brain, which we are going to investigate using the method described in this report.

## Synthesis

The decision was a result of the Reviewing Editor Marlene Bartos and the peer reviewers coming together and discussing their recommendations until a consensus was reached. A fact-based synthesis statement explaining their decision and outlining what is needed to prepare a revision is listed below. The following reviewers agreed to reveal their identity: James Bourne, Peer Wulff

Synthesis of Reviews:

Reviewer 1

Major comments:

1. The authors obtained two-photon images during the first week after surgery and calculate some basic in vivo spine dynamics. However, as they comment themselves in the Technical considerations section (page 13), the surgery itself is quite invasive and can perturb the brain tissue. It was demonstrated in mice by Holtmaat et al (Nat Protoc. 2009; 4(8): 1128-1144) that craniotomy induces a significant increase of reactive astrocytes under the craniotomized area lasting for at least two weeks after surgery. It is therefore questionable whether the measured spine dynamics are physiologically relevant. We would suggest first assessing the physiological status of the brain and providing data on brain integrity and presence of glial cells before showing measurements of spine dynamics.

2. Since this is a methods/new tools type of article, more detail on the optical window creation should be provided. For example:

- mention any use of analgesic, antibiotic and anti-inflammatory drugs and their frequency of application.

- what equipment was used to reopen/enlarge the hole in the skull?

- what does deflection of the dura entail? Is it a complete resection? If so, how large was the area that was resected?

- what are the dimensions of the coverglass? Was it located under or on top of the skull?

- it would be helpful to include more pictures of the creation of the optical window, including the attachment of the metal plate.

- the authors should include a comment on the clarity of the optical window over the course of time after surgery.

3. The authors make a comparison between spine densities in vivo and in fixed tissue using two different labelling methods. The fixed dye injected tissue however seems to come from an animal that has not been subjected to craniotomy. Given the fact that craniotomy can temporarily influence brain integrity, this comparison is not really relevant. Also we would suggest making a direct comparison of the same dentritic structures in vivo and ex vivo rather than inferring conclusions by indirect comparison.

Minor comments:

1. Please have article reviewed by native English speaker in order to increase reading fluency. Also, the word 'technique' is repeatedly used in the text and refers to either imaging techniques or labelling techniques and it is not always immediately clear which one is meant.

2. page 1, line 4: change appropriate techniques into appropriate labelling techniques.

3. page 1, line 16: change new technique into new labelling techniques.

4. page 1, section Significance Statement: consider rephrasing first 6 lines to increase readability.

5. page 2, line 4-5: change Our technique into Our approach.

6. page 2, line 5-6: change This technique into This labelling technique.

7. page 5, section Virus injection:

- did the animals receive any analgesic after surgery?

- was the hole in the skull closed after the virus injection?

- how long were the animals on antibiotics or anti-inflammatory drugs for after injury?

8. page 7, section Immunohistochemistry: provide details on how the brains were sectioned and on section thickness

9. page 9, line 15: change AAV:TRE-hrGFP. into AAV:TRE-hrGFP in marmosets.

10. page 10, line 14: mention from how many different dendrites images were acquired.

11. page 10, line 16: define turnover rate, or give formula in methods section (turnover rate is usually number of gains + number of losses divided by two times the initial number of spines). Is the graph in Figure 4D showing fractional gains and losses rather than turnover rates (i.e. number of gains or losses divided by initial number of spines)? It is mentioned that the turnover rate is similar to what has been described in other studies. Provide actual numbers either in text or table format. The mean and SD values that are mentioned on line 15, are these based on calculations per dendrite?

12. page 10, line 19: change shape of spines into spine length.

13. page 11, line 1: title is grammatically incorrect and doesn't not make sense.

14. page 12, line 3: mention the number of dendrites you imaged from. Are the densities displayed in Figure 5 calculated per dendrite?

15. page 12: line 6: change we did not observe weaker signals into we were not able to detect weaker signals.

16. page 13, lines 17-19: rephrase sentences (improve English and omit repetition of the word observe).

17. page 20, legend Figure 2B: clarify if the pictures are maximum projections, and if so, what the stack height (or number of slices) is for each of the three conditions.

18. page 20, line 12: change difference in cell density into difference in labelled cell density.

19. page 20, line 18: change color of letter I in Image.

20. page 21, legend Figure 4: mention how many days after craniotomy these images were obtained.

Reviewer 2:

This study describes the use of an intersectional viral approach to achieve sparse GFP labeling of neurons in marmoset neocortex for in vivo 2 photon spine imaging. The study describes spine dynamics during repetitive imaging and compares in vivo data with data from fixed tissue after GFP IHC and Dye loading. In particular the comparison of data from in vivo and ex vivo imaging and is interesting. In general the paper is clearly written and informative. However, sparse neuronal labeling could possibly have been achieved simply by reducing the titer of a conventional GFP virus.

Minor points:

The information on anesthesia is fragmented. Were different protocols used for anesthesia during perfusion for Dye injection and IHC? How was anesthesia induced (ketamine/xylazine or isoflurane) and maintained (isoflurane?) during viral injections?

Very little information is given regarding the injection sites. What were the stereotaxic coordinates?

The sequence of figures 1 and 2 should be changed according to their mentioning in the text.

p. 10, l 14: "...we analyzed 779 spines (12 sites, 3 animals, total dendrite length of 2238 micrometers)..." - what exactly is meant by 12 sites? How far are these sites apart? Does each site represent a different neuron or different segments on the same neuron?

Fig. 4, D: What do the antennas show in the box plot?

## References

[B1] Ako R, Wakimoto M, Ebisu H, Tanno K, Hira R, Kasai H, Matsuzaki M, Kawasaki H (2011) Simultaneous visualization of multiple neuronal properties with single-cell resolution in the living rodent brain. Mol Cell Neurosci 48:246–257. 10.1016/j.mcn.2011.08.005 21884798

[B2] Ballesteros-Yáñez I, Benavides-Piccione R, Elston GN, Yuste R, DeFelipe J (2006) Density and morphology of dendritic spines in mouse neocortex. Neuroscience 138:403–409. 10.1016/j.neuroscience.2005.11.038 16457955

[B3] Bernard A, Lubbers LS, Tanis KQ, Luo R, Podtelezhnikov AA, Finney EM, McWhorter MM, Serikawa K, Lemon T, Morgan R, Copeland C, Smith K, Cullen V, Davis-Turak J, Lee CK, Sunkin SM, Loboda AP, Levine DM, Stone DJ, Hawrylycz MJ, et al. (2012) Transcriptional architecture of the primate neocortex. Neuron 73:1083–1099. 10.1016/j.neuron.2012.03.002 22445337 PMC3628746

[B4] Caroni P (1997) Overexpression of growth-associated proteins in the neurons of adult transgenic mice. J Neurosci Methods 71:3–9. 9125370 10.1016/s0165-0270(96)00121-5

[B5] Denk W, Strickler JH, Webb WW (1990) Two-photon laser scanning fluorescence microscopy. Science 248:73–76. 2321027 10.1126/science.2321027

[B6] Denk W, Svoboda K (1997) Photon upmanship: why multiphoton imaging is more than a gimmick. Neuron 18:351–357. 9115730 10.1016/s0896-6273(00)81237-4

[B7] Elston GN, Benavides-Piccione R, DeFelipe J (2005) A study of pyramidal cell structure in the cingulate cortex of the macaque monkey with comparative notes on inferotemporal and primary visual cortex. Cereb Cortex 15:64–73. 10.1093/cercor/bhh109 15238445

[B8] Elston GN, Rockland KS (2002) The pyramidal cell of the sensorimotor cortex of the macaque monkey: phenotypic variation. Cereb Cortex 12:1071–1078. 12217971 10.1093/cercor/12.10.1071

[B9] Elston GN, Tweedale R, Rosa MGP (1999) Cellular heterogeneity in cerebral cortex: a study of the morphology of pyramidal neurones in visual areas of the marmoset monkey. J Comp Neurol 415:33–51. 10540356 10.1002/(sici)1096-9861(19991206)415:1<33::aid-cne3>3.0.co;2-m

[B10] Feng G, Mellor RH, Bernstein M, Keller-Peck C, Nguyen QT, Wallace M, Nerbonne JM, Lichtman JW, Sanes JR (2000) Imaging neuronal subsets in transgenic mice expressing multiple spectral variants of GFP. Neuron 28:41–51. 11086982 10.1016/s0896-6273(00)00084-2

[B11] Fu M, Yu X, Lu J, Zuo Y (2012) Repetitive motor learning induces coordinated formation of clustered dendritic spines in vivo. Nature 483:92–95. 10.1038/nature10844 22343892 PMC3292711

[B12] Grutzendler J, Kasthuri N, Gan WB (2002) Long-term dendritic spine stability in the adult cortex. Nature 420:812–816. 10.1038/nature01276 12490949

[B13] Hioki H, Kuramoto E, Konno M, Kameda H, Takahashi Y, Nakano T, Nakamura KC, Kaneko T (2009) High-level transgene expression in neurons by lentivirus with Tet-Off system. Neurosci Res 63:149–154. 10.1016/j.neures.2008.10.010 19028532

[B14] Holtmaat AJ, Trachtenberg JT, Wilbrecht L, Shepherd GM, Zhang X, Knott GW, Svoboda K (2005) Transient and persistent dendritic spines in the neocortex in vivo. Neuron 45:279–291. 10.1016/j.neuron.2005.01.003 15664179

[B15] Ji Y, Lu Y, Yang F, Shen W, Tang TT, Feng L, Duan S, Lu B (2010) Acute and gradual increases in BDNF concentration elicit distinct signaling and functions in neurons. Nat Neurosci 13:302–309. 10.1038/nn.2505 20173744 PMC4780419

[B16] Kim SK, Nabekura J (2011) Rapid synaptic remodeling in the adult somatosensory cortex following peripheral nerve injury and its association with neuropathic pain. J Neurosci 31:5477–5482. 10.1523/JNEUROSCI.0328-11.2011 21471384 PMC6622722

[B17] Komatsu Y, Watakabe A, Hashikawa T, Tochitani S, Yamamori T (2005) Retinol-binding protein gene is highly expressed in higher-order association areas of the primate neocortex. Cereb Cortex 15:96–108. 10.1093/cercor/bhh112 15217901

[B18] Konishi M, Kawamoto K, Izumikawa M, Kuriyama H, Yamashita T (2008) Gene transfer into guinea pig cochlea using adeno-associated virus vectors. J Gene Med 10:610–618. 10.1002/jgm.1189 18338819

[B19] Niu Y, Yu Y, Bernat A, Yang S, He X, Guo X, Chen D, Chen Y, Ji S, Si W, Lv Y, Tan T, Wei Q, Wang H, Shi L, Guan J, Zhu X, Afanassieff M, Savatier P, Zhang K, et al. (2010) Transgenic rhesus monkeys produced by gene transfer into early-cleavage-stage embryos using a simian immunodeficiency virus-based vector. Proc Natl Acad Sci U S A 107:17663–17667. 10.1073/pnas.1006563107 20870965 PMC2955145

[B20] Oga T, Aoi H, Sasaki T, Fujita I, Ichinohe N (2013) Postnatal development of layer III pyramidal cells in the primary visual, inferior temporal, and prefrontal cortices of the marmoset. Front Neural Circuits 7:31. 10.3389/fncir.2013.00031 23483808 PMC3592264

[B21] R Development Core Team (2012) R: a language and environment for statistical computing. Vienna, Austria: R Foundation for Statistical Computing.

[B22] Sasaki E, Suemizu H, Shimada A, Hanazawa K, Oiwa R, Kamioka M, Tomioka I, Sotomaru Y, Hirakawa R, Eto T, Shiozawa S, Maeda T, Ito M, Ito R, Kito C, Yagihashi C, Kawai K, Miyoshi H, Tanioka Y, Tamaoki N, et al. (2009) Generation of transgenic non-human primates with germline transmission. Nature 459:523–527. 10.1038/nature08090 19478777

[B23] Sasaki T, Komatsu Y, Watakabe A, Sawada K, Yamamori T (2010) Prefrontal-enriched SLIT1 expression in old world monkey cortex established during the postnatal development. Cereb Cortex 20:2496–2510. 10.1093/cercor/bhp319 20123755 PMC2936805

[B24] Stettler DD, Yamahachi H, Li W, Denk W, Gilbert CD (2006) Axons and synaptic boutons are highly dynamic in adult visual cortex. Neuron 49:877–887. 10.1016/j.neuron.2006.02.018 16543135

[B25] Takahata T, Komatsu Y, Watakabe A, Hashikawa T, Tochitani S, Yamamori T (2006) Activity-dependent expression of occ1 in excitatory neurons is a characteristic feature of the primate visual cortex. Cereb Cortex 16:929–940. 10.1093/cercor/bhj034 16151175

[B26] Takaji M, Komatsu Y, Watakabe A, Hashikawa T, Yamamori T (2009) Paraneoplastic antigen-like 5 gene (PNMA5) is preferentially expressed in the association areas in a primate specific manner. Cereb Cortex 19:2865–2879. 10.1093/cercor/bhp062 19366867 PMC2774394

[B27] Trachtenberg JT, Chen BE, Knott GW, Feng G, Sanes JR, Welker E, Svoboda K (2002) Long-term in vivo imaging of experience-dependent synaptic plasticity in adult cortex. Nature 420:788–794. 10.1038/nature01273 12490942

[B28] Vidal M, Morris R, Grosveld F, Spanopoulou E (1990) Tissue-specific control elements of the Thy-1 gene. EMBO J 9:833–840. 1968831 10.1002/j.1460-2075.1990.tb08180.x PMC551743

[B29] Watakabe A, Komatsu Y, Sadakane O, Shimegi S, Takahata T, Higo N, Tochitani S, Hashikawa T, Naito T, Osaki H, Sakamoto H, Okamoto M, Ishikawa A, Hara SI, Akasaki T, Sato H, Yamamori T (2009) Enriched expression of serotonin 1B and 2A receptor genes in macaque visual cortex and their bidirectional modulatory effects on neuronal responses. Cereb Cortex 19:1915–1928. 10.1093/cercor/bhn219 19056862 PMC2705701

[B30] Watakabe A, Takaji M, Kato S, Kobayashi K, Mizukami H, Ozawa K, Ohsawa S, Matsui R, Watanabe D, Yamamori T (2014) Simultaneous visualization of extrinsic and intrinsic axon collaterals in Golgi-like detail for mouse corticothalamic and corticocortical cells: a double viral infection method. Front Neural Circuits 8:110. 10.3389/fncir.2014.00110 25278843 PMC4166322

[B31] Xu HT, Pan F, Yang G, Gan WB (2007) Choice of cranial window type for in vivo imaging affects dendritic spine turnover in the cortex. Nat Neurosci 10:549–551. 10.1038/nn1883 17417634

[B32] Yamahachi H, Marik SA, McManus JN, Denk W, Gilbert CD (2009) Rapid axonal sprouting and pruning accompany functional reorganization in primary visual cortex. Neuron 64:719–729. 10.1016/j.neuron.2009.11.026 20005827 PMC2818836

[B33] Yamamori T (2011) Selective gene expression in regions of primate neocortex: implications for cortical specialization. Prog Neurobiol 94:201–222. 10.1016/j.pneurobio.2011.04.008 21621585

[B34] Yamamori T, Rockland KS (2006) Neocortical areas, layers, connections, and gene expression. Neurosci Res 55:11–27. 10.1016/j.neures.2006.02.006 16546282

[B35] Yu H, Majewska AK, Sur M (2011) Rapid experience-dependent plasticity of synapse function and structure in ferret visual cortex in vivo. Proc Natl Acad Sci 108:21235–21240. 10.1073/pnas.1108270109 22160713 PMC3248533

[B36] Zuo Y, Lin A, Chang P, Gan WB (2005) Development of long-term dendritic spine stability in diverse regions of cerebral cortex. Neuron 46:181–189. 10.1016/j.neuron.2005.04.001 15848798

